# The role of full-length apoE in clearance of Gram-negative bacteria and their endotoxins

**DOI:** 10.1016/j.jlr.2021.100086

**Published:** 2021-05-18

**Authors:** Ganna Petruk, Malin Elvén, Erik Hartman, Mina Davoudi, Artur Schmidtchen, Manoj Puthia, Jitka Petrlova

**Affiliations:** 1 Division of Dermatology and Venereology, Institution of Clinical Sciences, Lund University, Lund, Sweden; 2 Division of Cancer and Infection Medicine, Institution of Clinical Sciences, Lund University, Lund, Sweden; 3 Department of Biomedical Sciences, Copenhagen Wound Healing Center, Bispebjerg Hospital, University of Copenhagen, Copenhagen, Denmark; 4 Division of Dermatology, Skane University Hospital, Lund, Sweden

**Keywords:** antimicrobial peptides, bacteria, host defense, innate immunity, infection, aggregation, lipopolysaccharide, lipid A, CD, λ_max_, maximum absorbance, AMP, antimicrobial peptide, LPS, lipopolysaccharide, LTA, lipoteichoic acid, PBS-T, PBS-Tween, RDA, radial diffusion assay, TEM, transmission electron microscopy, TH, Todd-Hewitt, VCA, viable count assay

## Abstract

ApoE is a well-known lipid-binding protein that plays a main role in the metabolism and transport of lipids. More recently, apoE-derived peptides have been shown to exert antimicrobial effects. Here, we investigated the antibacterial activity of apoE using in vitro assays, advanced imaging techniques, and in vivo mouse models. The formation of macromolecular complexes of apoE and endotoxins from Gram-negative bacteria was explored using gel shift assays, transmission electron microscopy, and CD spectroscopy followed by calculation of the α-helical content. The binding affinity of apoE to endotoxins was also confirmed by fluorescent spectroscopy detecting the quenching and shifting of tryptophan intrinsic fluorescence. We showed that apoE exhibits antibacterial activity particularly against Gram-negative bacteria such as *Pseudomonas aeruginosa* and *Escherichia coli*. ApoE protein folding was affected by binding of bacterial endotoxin components such as lipopolysaccharide (LPS) and lipid A, yielding similar increases in the apoE α-helical content. Moreover, high-molecular-weight complexes of apoE were formed in the presence of LPS, but not to the same extent as with lipid A. Together, our results demonstrate the ability of apoE to kill Gram-negative bacteria, interact with their endotoxins, which leads to the structural changes in apoE and the formation of aggregate-like complexes.

## Graphical abstract

**Figure:**
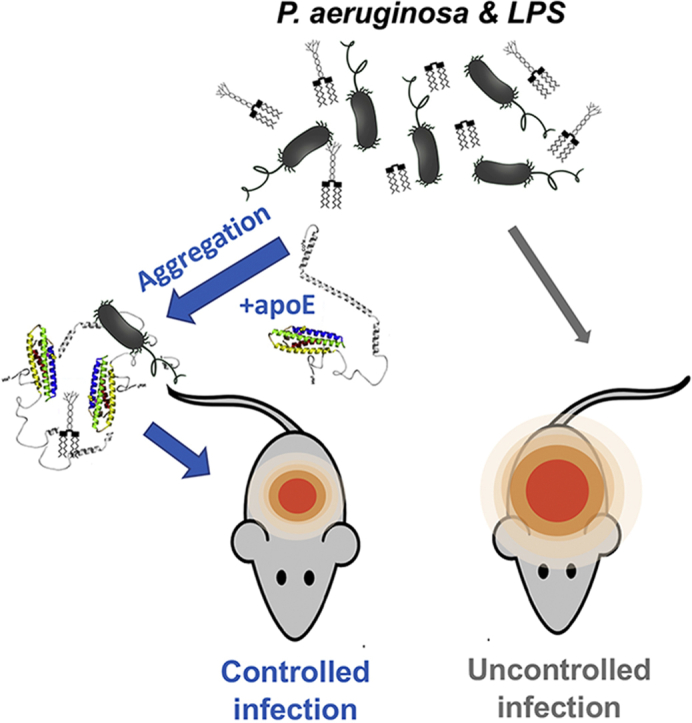

ApoE, a 34 kDa heterodimeric glycoprotein, is a well-known lipid-binding protein that plays a main role in the metabolism and transport of lipids (1). ApoE is primarily produced by the liver and macrophages in the peripheral tissues and by astrocytes in the central nervous system, where apoE plays a critical role in cholesterol homeostasis (2). In humans, apoE occurs in three isoforms: E2, E3, or E4 (3). Although the isoforms differ from each other by just one or two amino acids at positions 112 and 158, their structures and functions vary widely (4, 5). E3 is the most common variant (78%) in the human population and has been well characterized in terms of structure and function (6, 7, 8). The E4 isoform (14%) has been linked to multiple diseases such as atherosclerosis, Alzheimer’s disease (AD), and multiple sclerosis (6, 9). The least common isoform is E2 (8%), which is connected to hyperlipidemia (6, 10).

Lipoprotein particles commonly constitute complexes known as HDL, IDL, VLDL, and chylomicrons, which are soluble in blood and able to bind soluble ligands or receptors through apolipoproteins that are present on the surface of the particles. Some apolipoproteins may be released from one type of the lipoprotein complex to another (exchangeable), and apoE is an exchangeable protein (11). Lipoproteins that are secreted into the bloodstream are considered to be a new pool of host defense molecules (12, 13).

More recently, various peptides from the receptor-binding region of apoE (amino acid residues 130–150), which is essential for its biological function in lipid metabolism, have been shown to exert both antiviral and antibacterial activity (14, 15, 16, 17). This region is responsible for the binding of apoE to the LDL receptor family, and also, within this sequence, residues 142–147 (the heparin-binding domain) mediate attachment of apoE to cell surfaces (18). In particular, some apoE-derived peptides were shown to be effective against multiresistant bacteria and reduced lipopolysaccharide (LPS)-induced storm of cytokines in THP-1 cells (16, 17). Therefore, in addition to their anti-infective activity, these peptides also show immunomodulatory activity (19). Two peptides from the same region were shown to prevent neurodegeneration and restore cognition in an AD model in *Drosophila melanogaster* (20), thus mimicking the protective effects of the full-length protein.

In this work, we hypothesized that the full-length version of apoE may have an impact on bacterial survival. Here, we demonstrated for the first time the antibacterial activity of full-length apoE in vitro and in vivo. Using advanced imaging techniques, we showed that bacteria aggregate in the presence of apoE. We also detected the formation of high-molecular-weight complexes of apoE after binding to bacterial endotoxins. This interaction provides a molecular explanation for the neutralizing effect of apoE on endotoxins.

## Materials and methods

### Bacterial strains

*Escherichia coli* (25922) and *Staphylococcus aureus* (29213) were purchased from the American Type Culture Collection. *S. aureus* SAP229 was kindly provided by Dr Roger Plaut (Division of Bacterial, Parasitic, and Allergenic Products, FDA, Bethesda, MD). *Pseudomonas aeruginosa* (PA01) was kindly provided by Dr B. Iglewski (University of Rochester), and *P. aeruginosa* XEN41 was purchased from PerkinElmer (Akron, OH).

### Endotoxins

LPS from *E. coli* (serotype 0111:B4, cat# L3024), LPS from *P. aeruginosa* 10 (cat# L8643), and lipoteichoic acid (LTA) from *S. aureus* (cat# tlrl-pslta) were purchased from Sigma-Aldrich. Lipid A from *E. coli* (serotype R515, cat# ALX-581-200-L002) was purchased form AH Diagnostics.

### Proteins and peptides

Human plasma apoE (cat# IHUAPOE) and human plasma apoA1 (cat# IRHPL0059) were purchased from Innovative Research. The thrombin-derived peptide GKY25 (GKYGFYTHVFRLKKWIQKVIDQFGE) (97% purity, acetate salt) was synthetized by AmbioPharm (Madrid, Spain).

### Animals

SKH-1 hairless and BALB/c male mice (Charles River Laboratories), 8–12 weeks old, were used for in vivo experiments. The animals were housed under standard conditions of light and temperature and had free access to standard laboratory chow and water.

### Viable count assay

Potential antibacterial activity of apoE on *E. coli*, *P. aeruginosa*, and *S. aureus* was explored by incubating one colony overnight in 5 ml of Todd-Hewitt (TH) medium. The next morning, the bacterial culture was refreshed and grown to the mid-logarithmic phase (absorbance at 620 nm of 0.4). The bacteria were then centrifuged, washed, and diluted 1:1,000 in 10 mM Tris buffer at pH 7.4 to obtain an approximate concentration of bacteria amounting to 2 × 10^6^ cfu/ml. Next, 50 μl of bacterial suspension was incubated with 1 and 5 μM of apoE, 5 μM of GKY25 (used as a positive control), or buffer control (10 mM Tris buffer at pH 7.4) for 2 h at 37°C. After 2 h, serial dilutions of the samples were plated on TH agar plates, incubated overnight at 37°C, and followed by colony counting the next day based on the following equation:

$$cfu/ml=\frac{colonies\;x\;dilution\;factor}{volume\;\left(per\;spot\right)on\;plate}$$

Antimicrobial activity of apoE was also assessed by the viable count assay (VCA) after preincubation for 30 min with 200 and 500 μg/ml heparin (Sigma-Aldrich). Four independent experiments were performed for each bacterial strain (21, 22).

### Radial diffusion assay

We used *E. coli*, *P. aeruginosa*, and *S. aureus* for the radial diffusion assay (RDA). The bacteria were grown to the mid-log phase in 10 ml of TH medium, spun down, washed, and suspended in 10 ml of 10 mM Tris buffer, pH 7.4, as for VCA. This step was followed by the addition of bacteria (4 × 10^6^ cfu) to 15 ml of under-lay agarose gel, consisting of 0.03% TH media, 1% (w/v) low-electroendosmosis-type agarose (Sigma-Aldrich), and 0.02% (v/v) Tween 20 (Sigma-Aldrich). The underlay was poured into a 144 mm diameter Petri dish. After solidification, 4 mm diameter wells were punched in the underlay, which were subsequently loaded with 6 μl of the buffer (negative control), 5 μM GKY25 (positive control), and apoE or apoAI in 10 mM Tris buffer at pH 7.4. The plates were thereafter incubated for 3 h at 37°C. Molten overlay gel (15 ml, 6% TH, and 1% low-electroendosmosis-type agarose in water) was added to the plate. We measured the antimicrobial activity of the peptides by measuring the radius of the clearing zone surrounding the wells after 18–24 h of incubation at 37°C (21, 22).

### Mouse model of subcutaneous infection

Overnight cultures of bioluminescent *P. aeruginosa* Xen41 or *S. aureus* 229 were refreshed and grown to the mid-logarithmic phase in TH media. Bacteria were washed (5.6 × 1,000 rpm, 15 min) and incubated with apoE (5 μM) for 2 h or injected directly without preincubation. A total of 200 μl of the mixture (1 × 10^6^ cfu/mouse) was injected subcutaneously into the dorsum of male SKH-1 hairless mice or shaved dorsum of BALB/c mice (8–12 weeks), which were anesthetized before injections, using a mixture of 2% isoflurane and oxygen. In vivo bacterial infection was then imaged by measuring bioluminescence in anesthetized mice in Vivo Imaging System (IVIS Spectrum, PerkinElmer Life Sciences), and the data obtained were analyzed using Living Image 4.0 Software (PerkinElmer). Five or six mice per treatment group were used. The animal model was previously described by Petrlova *et al.* and Puthia *et al.* (21, 22).

### Blue native PAGE and Western blot

Twenty-one microliters of ApoE (5 μM) was mixed with either 10 mM Tris as control or endotoxins (200 μg/ml final concentration). Samples were incubated for 30 min at 37°C before mixing with the loading buffer (4× loading buffer native gel, cat#BN2003, Life Technologies), and subsequent 28 μl was loaded onto 4–16% Bis-Tris Native Gels (cat#BN1002BOX, Life Technologies). Control experiments with 5 μM apoA1 and apoE were performed with or without 100 μg/ml LPS from *E. coli* and loaded onto gel after a 30-min incubation at 37°C. Samples were run in parallel with a marker (NativeMark Unstained Protein Standard, cat#LC0725, Life Technologies) at 150 V for 100 min. Gels were run in duplicates for each experiment: one for gel analysis after destaining from Coomassie and subsequent staining with GelCode Blue Safe Protein (cat# 1860983, Thermo Scientific), while the other was transferred to a 0.2 μm polyvinylidene fluoride membranes (Trans-Blot Turbo Transfer Pack, cat #1704156, Bio-Rad) via a Trans Turbo Blot system (Bio-Rad). Thereafter, the membrane was destained with 70% ethanol and blocked with 5% milk in 1× PBS-Tween (PBS-T) for 30 min at room temperature. The membrane was incubated with mouse mAb anti-human apoE (cat#ab1906, Abcam), at a concentration of 1 μg/ml diluted in 1% fat-free milk in 1× PBS-T, overnight at 4°C. ApoE and its high-molecular-weight complexes were then detected using a secondary rabbit anti-mouse polyclonal antibody that was conjugated to HRP (cat#P0260, Dako) (diluted 1:1,000 in 1× PBS-T complemented with 5% milk) after incubation for 60 min at room temperature. PBS-T was used to wash the membrane after each step (3 × 10 min), and the last wash after the secondary antibody was performed five times. The bands were revealed by incubating the membrane in the developing substrate (Super Signal West Pico PLUS Chemiluminescent Substrate, cat#34580, Thermo Scientific). Signal was acquired by a ChemiDoc (Bio-Rad) system. All the experiments were performed at least three times (21).

### CD

CD measurements were performed using a Jasco J-810 spectropolarimeter equipped with a Jasco CDF-426S Peltier that was set to 25°C. Protein was diluted to 5 μM in 10 mM Tris at pH 7.4 and incubated with or without 200 μg/ml LPS from *E. coli* or *P. aeruginosa*, Lipid A from *E. coli*, LTA from *S. aureus*, or heparin (200 and 500 μg/ml). Measurements were performed after a 1–5 min or a 30 min incubation at room temperature in a 0.1 cm quartz cuvette. Scans were measured over the far-UV wavelength interval 200–260 nm, with a scan speed of 20 nm/min. An average of five scans for each sample was collected. The baseline (10 mM Tris buffer alone or with different ligands) was subtracted from the spectrum of each sample for normalization.

The α-helical content was calculated according to previously published equations (23) and reported below:

$$\%\;\text{α}-helical\;content\;=\;\frac{\left(\left[\theta \right]222+3000\right)}{\left(-39000+3000\right)}$$

Where −3,000 and −39,000 have been established previously as constants based on the helicity of poly-L-lysine as described by (24):

$$\left[\Theta \right]\;Molar\;ellipticity\;=\;\frac{\left(\theta 222\right)\;\times \;\left(MRW\right)}{\left(10\;\times \;c\;\times \;l\right)\;}$$

Where θ_222_ = observed ellipticity at 222 nm in millidegrees, c = concentration in g/l, l = path length of the cuvette (cm), and MRW = mean residual weight, that is, molecular weight of the protein (Da)/(number of amino acids). All the experiments were performed at least three times (21, 25).

### Transmission electron microscopy

*E. coli* and *P. aeruginosa* were visualized using transmission electron microscopy (TEM) (Jeol JEM 1230; Jeol, Japan) in combination with negative staining after incubation with apoE (5 μM) or buffer as described in the VCA method. Images of endotoxins (200 μg/ml) in the presence or absence of apoE (5 μM) were taken after incubation for 120 min at 37°C. For the mounted samples, 10 view fields were examined on the grid (pitch 62 μm) from three independent sample preparations. Samples were adsorbed onto carbon-coated grids (Copper mesh, 400) for 60 s and stained with 7 μl of 2% uranyl acetate for 30 s. The grids were rendered hydrophilic via glow discharge at low air pressure (21, 25). The size of aggregates was analyzed as the mean of gray value/μm ± SD by ImageJ 1.52k, after all the images were converted to 8 bit and the threshold was manually adjusted.

### Fluorescence microscopic analysis of live/dead bacteria

*E. coli* and *P. aeruginosa* viability in the aggregates was assessed by using LIVE/DEAD® BacLight^TM^ Bacterial Viability Kit (Invitrogen, Molecular Probes, Carlsbad, CA). Bacterial suspensions were prepared as described above for the VCA. Strains were treated with 5 μM apoE, 5 μM GKY25, or 10 mM Tris at pH 7.4. After a 1 h incubation at 37°C, samples were mixed 1:1 with the dye mixture, followed by incubation for 15 min in the dark at room temperature. The dye mixture was prepared according to the manufacturer's protocol, that is, 1.5 μl of component A (SYTO 9 green-fluorescent nucleic acid stain) and 1.5 μl of component B (red-fluorescent nucleic acid stain propidium iodide) were dissolved in 1 ml of 10 mM Tris at pH 7.4. Five microliters of stained bacterial suspension was trapped between a slide and an 18 mm square coverslip. Ten view fields (1 × 1 mm) were examined from three independent sample preparations using a Zeiss AxioScope A.1 fluorescence microscope (objectives: Zeiss EC Plan-Neofluar 100×/1.3 oil and 40×; camera: Zeiss AxioCam MRm; acquisition software: Zeiss Zen 2.6 [blue edition]) (21).

### In silico prediction of antimicrobial peptides

ApoE was scanned for antimicrobial peptides (AMPs) using two in silico predictors: AmpGram (26) and a method for detection of “cryptic” AMPs, utilizing amino acid properties and peptide length developed by Pane *et al.* (27). AmpGram is an AMP predictor, utilizing n-grams and a random forest classifier to predict AMPs with high accuracy while allowing for high-throughput proteomic screening. The AmpGram package (https://cran.r-project.org/web/packages/AmpGram/index.html) was used in R 4.0.2 (in the RStudio 1.3.1073 IDE). The model was applied to the FASTA sequence of apoE fetched from UniProt (P02649), and the results were visualized using the AmpGram package.

Thereafter, the sequence was inserted into Microsoft Excel containing the algorithm presented by Pane *et al.* (REF) using the values *m* = 0.700 and n = 0.800. The helical property of the peptide with the highest antimicrobial score was visualized using NetWheels (28). ApoE and the predicted AMPs were visualized in the NMR structure of human apoE (PDB accession 2L7B, https://www.rcsb.org/structure/2L7B) as a 3D model using PyMOL 2.3.4 (29).

### Fluorescence spectroscopy

The emission fluorescence spectra of tryptophan in apoE were measured between 300 and 450 nm, after excitation at 280 nm. Intrinsic fluorescence of 5 μM apoE (10 mM Tris at pH 7.4) was measured in a 3×3 mm quartz cuvette using a Jasco J-810 spectropolarimeter equipped with an FMO-427S fluorescence module, a scan speed of 200 nm/min, and a 2-nm slit width. The interactions between apoE and bacterial ligands were performed by measuring intrinsic fluorescence of protein at 25°C, immediately after addition of increasing concentrations of ligands (0–100 μg/ml). Then, the dissociation constant (*K*_*d*_) was calculated from the spectral shift of maximum absorbance (λ_max_) as a function of the concentration of the bacterial ligands. The signal obtained for the protein alone was subtracted from all spectra. The results are expressed as an average of three independent experiments ± SEM (30).

### Statistical analysis

The graphs of the VCA, *K*_*d*_ (from fluorescence spectroscopy), and α-helical content (from CD measurements) are presented as the mean ± SD (VCA and α-helical content) or SEM (*K*_*d*_) from at least three independent experiments. We assessed differences in these assays using the Brown-Forsythe ANOVA test for CD and one-way ANOVA with Dunnett’s multiple comparison tests for the VCA and *K*_*d*_. All data were analyzed using GraphPad Prism (GraphPad Software, Inc.). In addition, *P*-values less than 0.05 were considered to be statistically significant (∗*P* < 0.05, ∗∗*P* < 0.01, ∗∗∗*P* < 0.001, and ∗∗∗∗*P* < 0.0001).

### Ethics statement

All animal experiments were performed according to the Swedish Animal Welfare Act SFS 1988:534 and were approved by the Animal Ethics Committee of Malmö/Lund, Sweden (permit numbers M88-91/14, M5934-19, M8871-19). Animals were kept under standard conditions of light and temperature and water ad libitum.

## Results

### Antibacterial activity of full-length apoE in vitro

**Fig. 1:**
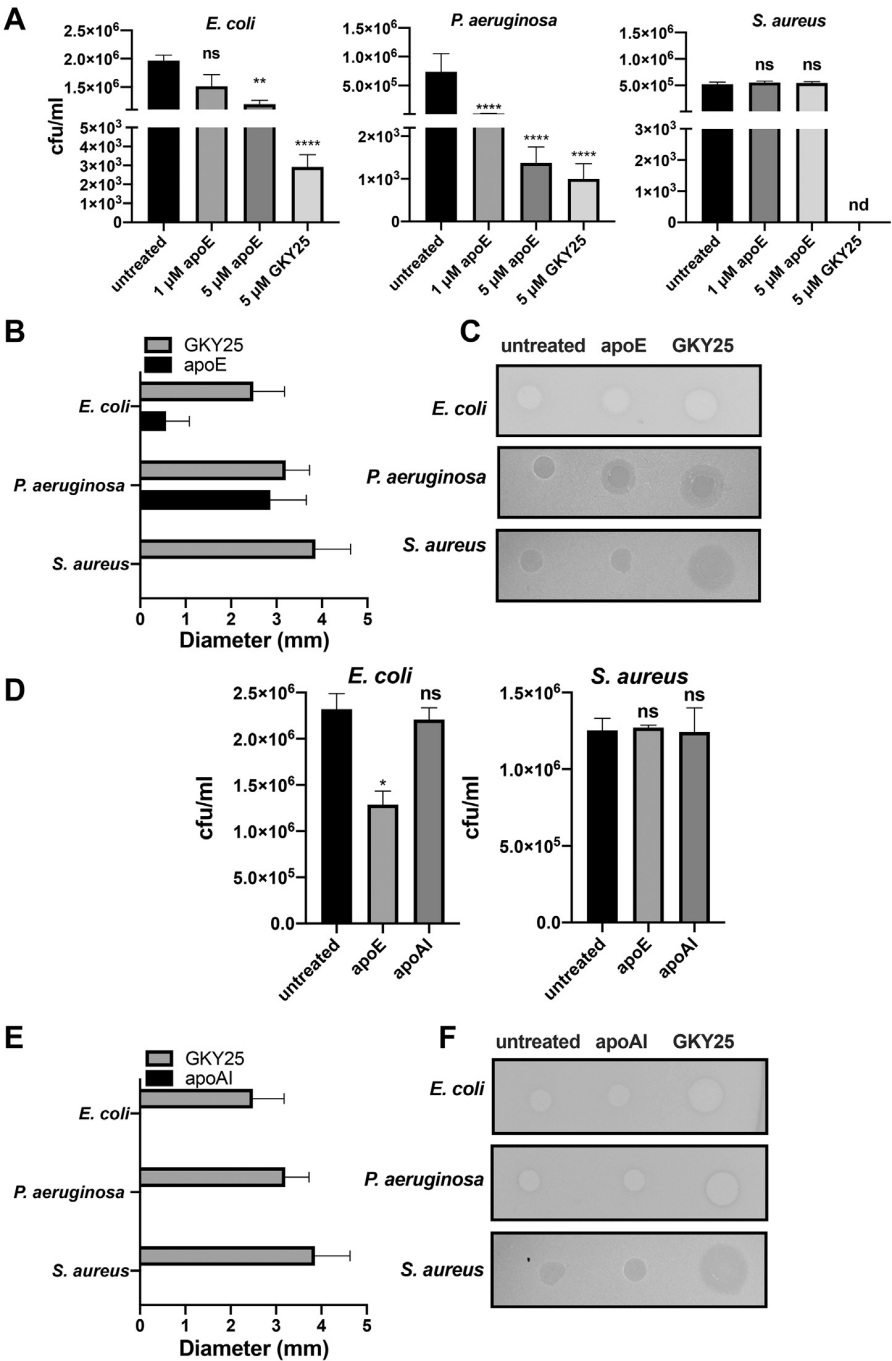
Antimicrobial activity of apoE in vitro. A: *Escherichia coli*, *Pseudomonas aeruginosa*, and *Staphylococcus aureus* were incubated with two different concentrations of apoE for 2 h, and then, the viable count assay was performed. Thrombin-derived peptide GKY25 was used as a positive control, whereas bacteria without any treatment were used as a negative control. Data are presented as the mean ± SEM of four independent experiments (n = 4). Statistical analysis was performed using a one-way ANOVA with Dunnett’s multiple comparison tests, ∗∗*P* ≤ 0.01, ∗∗∗∗*P* ≤ 0.0001. B, C: *E. coli*, *P. aeruginosa*, and *S. aureus* were treated with 5 μM apoE, 5 μM GKY25, or buffer only. The next day, the diameter of the clearance zones was measured. The data are presented as the mean ± SEM (n = 3) (B). One representative image of RDA is shown (C). D: *E. coli* and *S. aureus* were treated with 5 μM of apoA1 or apoE for 2 h, and then the viable count assay was performed. Data are presented as the mean ± SEM (n = 3). Statistical analysis was performed using a one-way ANOVA with Dunnett’s multiple comparison tests, ∗*P* ≤ 0.05. E, F: RDA of *E. coli*, *P. aeruginosa*, and *S. aureus* after treatment with 5 μM apoA1, 5 μM GKY25, or buffer. The diameter of the clearance zones is reported in panel E. Data are presented as the mean ± SEM (n = 3). Statistical analysis was performed using a one-way ANOVA with Dunnett’s multiple comparison tests, ∗*P* ≤ 0.05. One representative image of RDA from three independent experiments is shown panel F. nd, not detected; ns, not significant; RDA, radial diffusion assay.

Full-length apoE that was derived from human plasma was analyzed for its potential antimicrobial effects against the two Gram-negative strains (*E. coli* and *P. aeruginosa*) and one Gram-positive strain (*S. aureus*) using the VCA (Fig. 1A). No significant reduction in the *E. coli* colony number was observed in the samples treated with 1 μM apoE. Upon incubation with 5 μM apoE, a slight but significant decrease in *E. coli* (around 2-fold cfu/ml) viability was detected. A significant reduction in bacterial counts of *P. aeruginosa* (by approximately 10^2^-fold cfu/ml) was obtained upon the addition of 1 μM apoE, which is the physiological concentration of protein in plasma. The antimicrobial effect of apoE was even more pronounced at a concentration of 5 μM (around 10^3^-fold cfu/ml). The bacterial killing efficiency of apoE against *P. aeruginosa* was comparable with that of the thrombin-derived anti-microbial peptide GKY25, which are used here as a positive control. *S. aureus* growth was not significantly affected by the same apoE concentrations. Moreover, we used a gel-based antimicrobial assay (RDA) to confirm the killing effect of 5 μM apoE on *E. coli* and *P. aeruginosa* by detecting zones of inhibition (Fig. 1B, C). ApoE (5 μM) did not inhibit the growth of *S. aureus* as detected in the above VCA (Fig. 1B, C). To validate the bacterial killing by specific apolipoproteins, we compared the results from VCA and RDA using apoE and apoA1 (primary protein component of HDL in plasma) on *E. coli*, *P. aeruginosa*, and *S. aureus* (Fig. 1D–F). ApoA1 yielded no significant antimicrobial activity on any of the bacterial strains. The antibacterial activity of apoE against *P. aeruginosa* was reduced 100-fold after addition of heparin (supplemental Fig. S1A). The interaction between apoE and heparin was confirmed by analyzing the changes in the secondary structure of the protein using CD spectroscopy (supplemental Fig. S1B).

### Antibacterial activity of full-length apoE in vivo

**Fig. 2:**
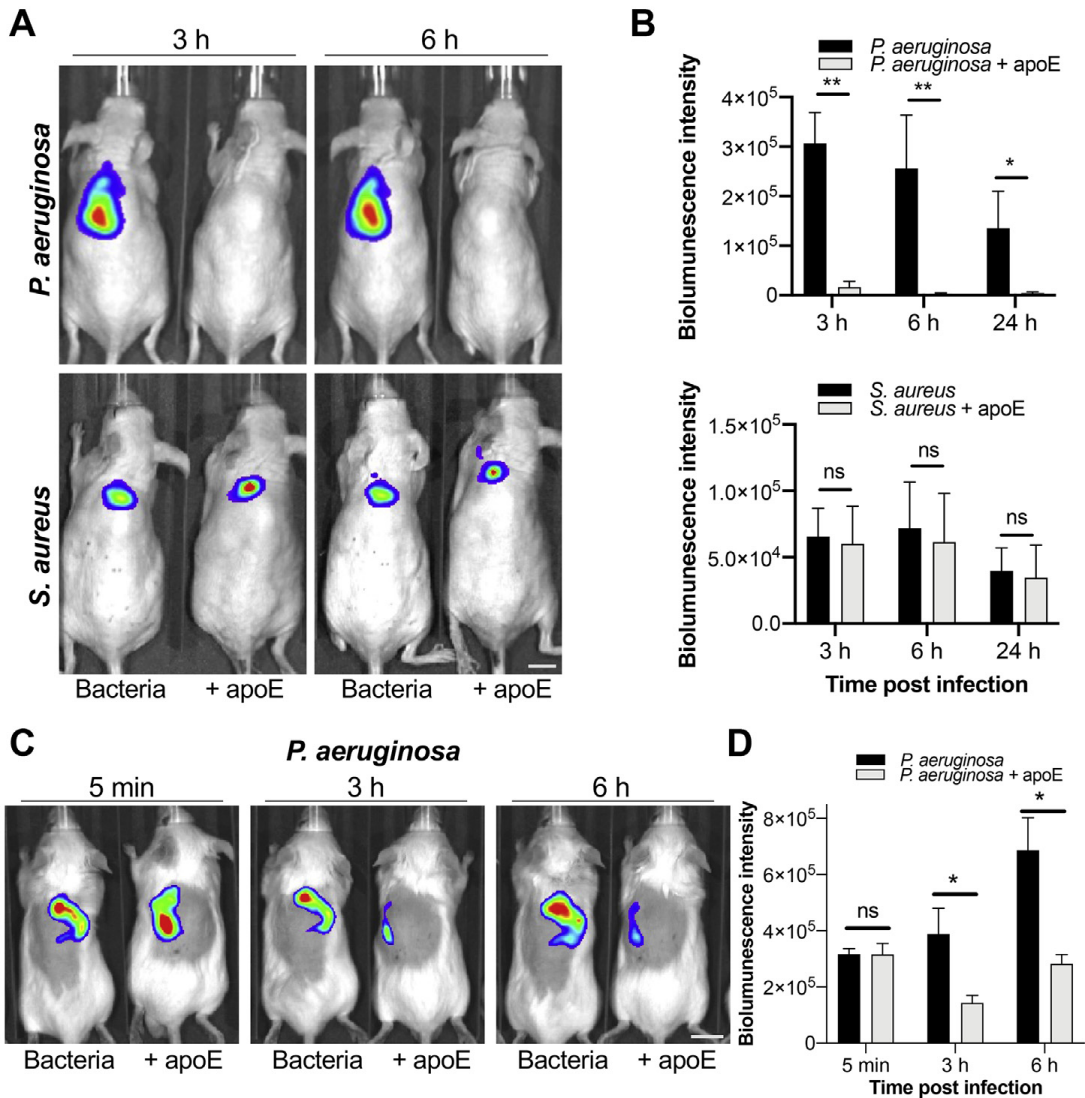
Antimicrobial activity of apoE in vivo. A: The effects of apoE in a mouse model of subcutaneous infection are illustrated. Bioluminescent *Pseudomonas aeruginosa* or *Staphylococcus aureus* (1 × 10^6^ cfu/mouse) were incubated for 2 h with 5 μM apoE or buffer only and then deposited subcutaneously in the dorsum of SKH-1 hairless male mice. Bioluminescence intensity was analyzed upon anesthesia using the IVIS bioimaging system. Representative images show bacterial luminescence at 3 and 6 h after infection. The scale bar represents 1 cm. B: The bar chart shows measured bioluminescence intensity emitted by the bacteria at 3, 6, and 24 h after infection. C: Bioluminescent bacteria (*P. aeruginosa*, 1 × 10^6^ cfu/mouse) were mixed with 5 μM apoE or buffer only and immediately deposited subcutaneously in the dorsum of BALB/c mice male mice. Bioluminescence intensity was analyzed upon anesthesia using the IVIS bioimaging system. Representative images show bacterial luminescence at 5 min and 3 and 6 h after infection. The scale bar represents 1 cm. D: the bar chart shows measured bioluminescence intensity emitted by the bacteria at 5 min and 3 and 6 h after infection. All in vivo data are presented as the mean ± SEM (n = 5–6 mice). ∗*P* ≤ 0.05, *∗∗P* ≤ 0.01; *P* values were determined using the Mann-Whitney *U* test. ns, not significant.

To further investigate the in vivo relevance of the effects of apoE, bioluminescent *P. aeruginosa* and *S. aureus* bacteria (10^6^ cfu/animal) were incubated with 5 μM apoE or buffer only and subcutaneously injected into SKH-1 hairless male mice. In this model, the bacterial dose that was used causes a transient and self-limiting infection. The results showed that apoE significantly reduced the bacterial load of *P. aeruginosa* after already 3 h, and the same effect was observed after 24 h as assessed by in vivo bioimaging (Fig. 2A–D). Mice injected with *P. aeruginosa* treated with the control buffer maintained their infection during this time period. No significant reduction of *S. aureus* growth was observed during the 24 h time period. The findings from the in vivo experiment were consistent with the in vitro data (Fig. 1A–C).

### ApoE induces aggregation of *P. aeruginosa* and *E. coli*

**Fig. 3:**
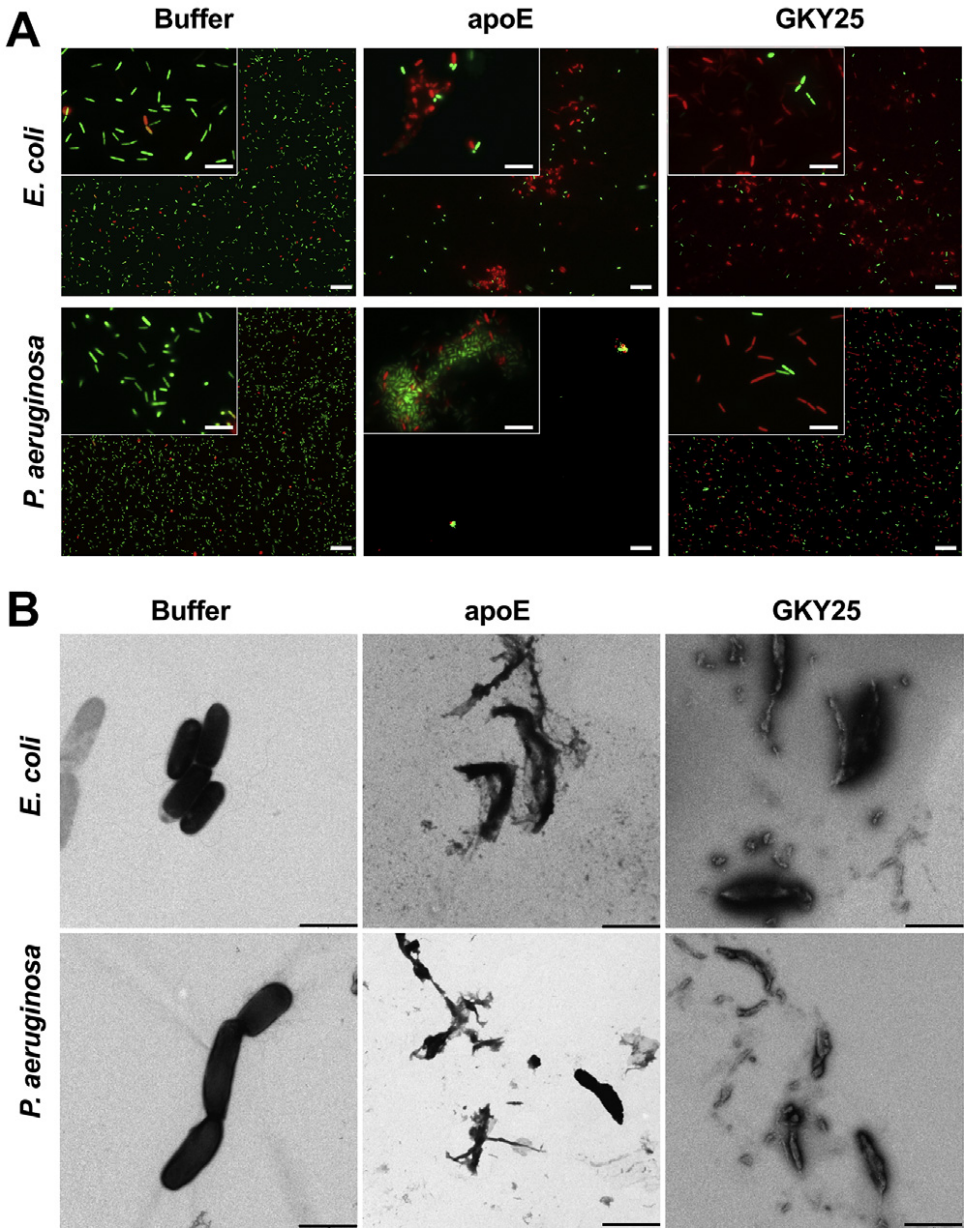
Visualization of *Escherichia coli* and *Pseudomonas aeruginosa* viability. A: LIVE/DEAD Viability Assay of Gram-negative bacteria stimulated with 5 μM apoE, 10 mM Tris buffer at pH 7.4 (negative control), or 5 μM GKY25 (positive control). Representable images from three independent experiments are presented (n = 3). At least 10 individual fields were acquired per each experiment. Live bacteria were stained with green SYTO 9 nucleic acid fluorescent dye, and dead bacteria were stained using red propidium iodine dye. The scale bar in the 100× magnification window (insert) represents 5 μm and 25 μm in the 40× window. B: *E. coli* and *P. aeruginosa* were treated with 5 μM apoE, buffer only (negative control), or 5 μM GKY25 (positive control) for 120 min, and then, the bacterial membrane morphology was analyzed by TEM. One representative image from three independent experiments is shown (10 individual fields per experiment, n = 3). The scale bar represents 1 μm. TEM, transmission electron microscopy.

In the same manner as for the VCA, *E. coli* and *P. aeruginosa* were incubated with 10 mM Tris buffer at pH 7.4 (negative control), 5 μM apoE, or GKY25 (positive control) followed by analysis of bacterial viability using a LIVE/DEAD Viability Assay (Fig. 3A). Epifluorescence microscopy analysis revealed that apoE treatment resulted in aggregation of both Gram-negative strains. For *P. aeruginosa*, mostly dead bacteria were detected in the aggregates. Clusters of dead bacteria were observed in the apoE-treated samples, where GKY25 killed single bacterial cells.

### ApoE induces morphological changes in bacterial membranes

Next, the killing effect of 5 μM apoE on *E. coli* and *P. aeruginosa* bacteria, as shown by the LIVE/DEAD assay, was evaluated using TEM (Fig. 3B). Bacteria that were treated with 10 mM Tris at pH 7.4 were used as a negative control, and GKY25 (5 μM) was used as the positive control. ApoE treatment induced severe morphological changes in bacteria, which was similar to the positive control GKY25. The disrupted morphology of the bacterial membrane was clearly visualized in both bacterial strains (Fig. 3B).

### In silico prediction of apoE-derived AMPs

**Fig. 4:**
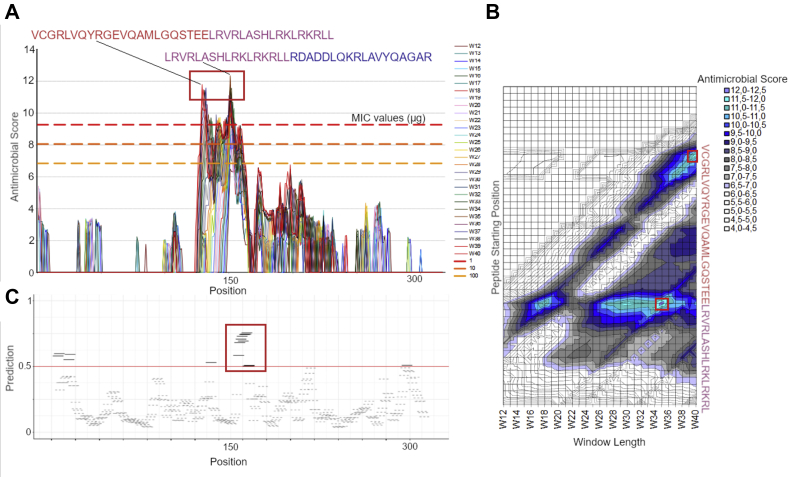
In silico prediction and visualization of AMPs. A: Proteomic screening for AMPs in apoE using peptide properties. The graph shows antimicrobial score for peptides of varying window lengths across the entire protein with i*n silico* minimum inhibitory concentration panel test values for 1, 10, and 100 μm. Two peaks can be seen around positions 130 and 150, resulting in the peptides VCGRLVQYRGEVQAMLGQSTEELRVRLASHLRKLRKRLLR and LRVRLASHLRKLRKRLLRDADDLQKRLAVYQAGAR, respectively. B: Map of antimicrobial score over the peptide length and starting position. Each point in the graph represents a peptide with the starting point labeled by the vertical axis and a window length as indicated by the horizontal axis. There are two distinct clusters generating antimicrobial peptides within apoE. These are situated around 130 and 150 and are generally >30 amino acids long. C: Proteomic screening of 10-mer AMPs in apoE using machine learning model (AmpGram). The model predicted 16 peptides to be classified as AMPs (according to the cutoff criterion of >0.5). The region around position 150 was predicted to most likely give rise to AMPs. AMPs, antimicrobial peptides.

Two in silico predictors were used on the apoE sequence to scan for AMP(s). First, an algorithm developed by Pane *et al.* was used (27). The algorithm results in an antimicrobial score based on the chemical properties of the given peptide such as charge, hydrophobicity, and length. Sliding windows ranging between 12 and 40 were used to scan the apoE sequence for AMPs. The results are shown in the graph (Fig. 4A) and map (Fig. 4B). As can be seen in Fig. 4B, the algorithm predicts two main regions to generate AMPs, situated at position 130 (VCGRLVQYRGEVQAMLGQSTEELRVRLASHLRKLRKRLLR) and 150 (LRVRLASHLRKLRKRLLRDADDLQKRLAVYQAGAR). The complete results are attached in supplementary information 2.

The second predictor used is called AmpGram (26). AmpGram is a novel predictor that utilizes a machine learning approach and was used to scan apoE for 10-mers. A sliding window of size 10 is used to generate overlapping peptides over the entire protein, whereafter the model outputs a predicted probability of each sequence's likeliness of being an AMP. The output value thus ranges between 0 and 1, and AmpGram inherently places a cutoff at 0.5. As can be seen in Fig. 4C, the region situated around position 150 was again predicted to generated AMPs. The sequence LRKLRKRLLR was given the highest probability of 0.7559. The complete results can be found in supplementary information 3.

As AMPs commonly possess a certain helical property, with strictly hydrophobic properties, the helical structure of common sequence LRVRLASHLRKLRKRLL was projected using NetWheels. The projection shows clearly divided regions of hydrophobic contra hydrophilic residues, which are typical amphipathic structural features of AMPs (supplemental Fig. S2A). Finally, the position of the peptides with the highest antimicrobial score (VCGRLVQYRGEVQAMLGQSTEELRVRLASHLRKLRKRLLR and LRVRLASHLRKLRKRLLRDADDLQKRLAVYQAGAR) in apoE was visualized using PyMOL (PDB accession 2L7B). The peptides overlap greatly and are situated in the center of apoE (supplemental Fig. S2B).

### Bacterial products induce the formation of macromolecular complexes with apoE

**Fig. 5:**
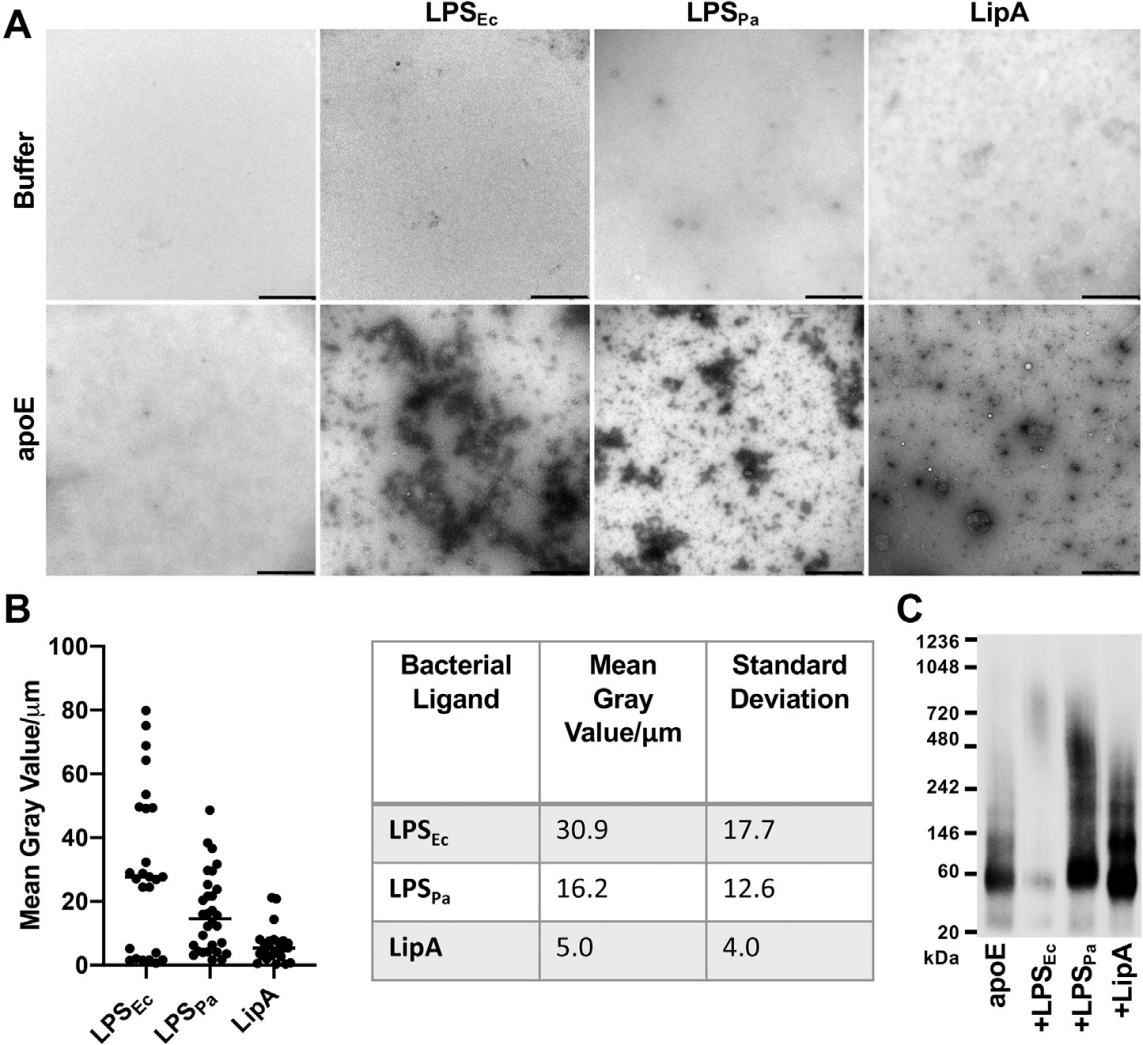
Visualization of macromolecular complexes of apoE and bacterial products. A, ApoE (5 μM) was incubated with 200 μg/ml LPS from *Escherichia coli* or *Pseudomonas aeruginosa* (denoted as LPS_Ec_ and LPS_Pa_, respectively) or with Lipid A from *E. coli* (LipA) for 60 min at 37°C. At the end of incubation, the macromolecular complexes were visualized by TEM. One representative image for each condition from three independent experiments is shown (n = 3). The scale bar represents 1 μm. B: Analysis of the complexes of apoE and ligands after TEM. Quantification was performed using ImageJ 1.52k after all the images were converted to 8 bit and the threshold was adjusted. The complexes of apoE and ligands are expressed as the mean of gray value/μm ± SD. In the graph, each point represents the measurement of one picture. The table summarizes the data from three independent experiments, after analysis of 10 pictures per each experiment (n = 3). C: ApoE (5 μM) was mixed with either Tris buffer or 200 μg/ml LPS from *E. coli* or *P. aeruginosa* (denoted as LPS_Ec_ and LPS_Pa_, respectively) or with Lipid A from *E. coli* (LipA). Samples were incubated for 30 min at 37°C and then analyzed by Western blot after Blue Native gel. One representative image from five independent experiments is shown (n = 5). LPS, lipopolysaccharide; TEM, transmission electron microscopy.

We investigated if apoE interacts with different toxic components of the Gram-negative bacteria cell wall such as LPS and lipid A, which both can cause septic shock when released to the bloodstream. Lipid A is the most toxic part of LPS, anchoring the molecule to the surface of bacteria. The protein was incubated with LPS (from *E. coli* and *P. aeruginosa*) or Lipid A (from *E. coli*) and then subsequently visualized by TEM-negative stain (Fig. 5A). TEM image analyses showed that apoE formed aggregates in the presence of LPS. The complexes with LPS appeared larger when compared with those that were generated with Lipid A. Moreover, the LPS source seemed to affect the aggregate size because the trend in the distribution of the mean complex area was larger in the samples that were challenged by LPS from *E. coli* than LPS from *P. aeruginosa* (Fig. 5B). Macromolecular complexes from the three individual experimental preparations are presented in supplemental Fig. S3A. The data obtained by TEM were confirmed by blue native PAGE and subsequent Western blot analysis against apoE (Fig. 5C). We detected a shift toward higher molecular complexes in the presence of all bacterial products. LPS from *E. coli* exhibited the lowest signal, possibly because of the formation of very high-molecular-weight complexes with apoE, which are not able to enter the Blue Native gel. To further demonstrate the specific interaction of apoE with LPS, we used apoA1 as a negative control in the Blue Native gel analysis, which did not show any change in the molecular weight after the LPS treatment (supplemental Fig. S3B).

### Structural changes of apoE upon interaction with endotoxins from Gram-negative bacteria

**Fig. 6:**
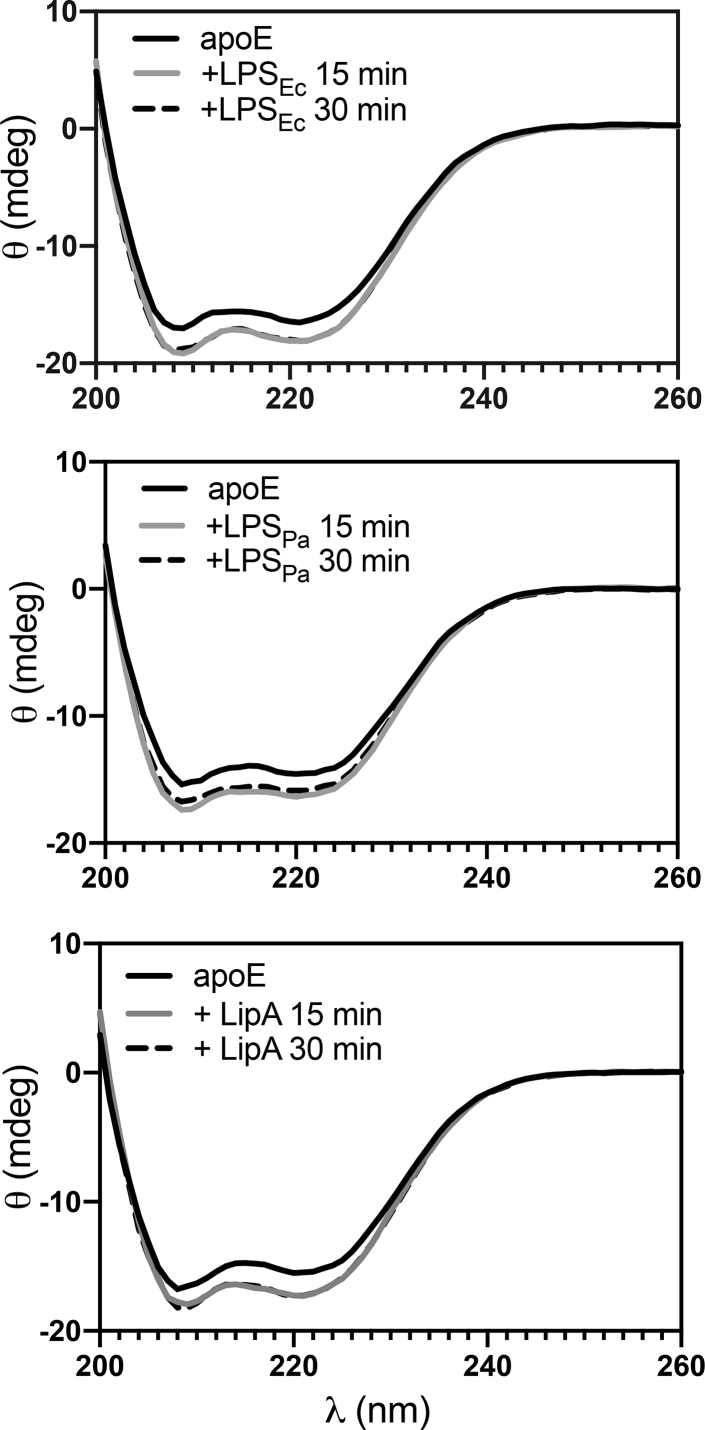
Change in the secondary structure of apoE is triggered by interaction with bacterial products. Far-UV CD spectra of 5 μM apoE alone or upon incubation with 200 μg/ml LPS from *Escherichia coli* (LPS_Ec_), LPS from *Pseudomonas aeruginosa* (LPS_Pa_), or Lipid A from *E. coli* (LipA) after 15 and 30 min. Figures show representative spectra from at least three independent experiments (n ≥ 3). LPS, lipopolysaccharide.

Ellipticity recordings in the far-UV spectra of apoE (5 μM) alone and in combination with different endotoxins (LPS and Lipid A from *E. coli* and LPS from *P. aeruginosa*) allowed measurement of changes in the secondary structure. The data obtained revealed an increase in the α-helical content of plasma apoE upon interaction with the ligands (Fig. 6). Investigation of a specific interaction between apoE and bacterial endotoxins was performed in an additional experiment where we used apoA1 as a negative control, which showed no change in the secondary structure of this protein (supplemental Fig. S3C). The α-helical content of apoE significantly increased after the challenge with bacterial products (LPS and Lipid A from *E. coli* and LPS from *P. aeruginosa*) (supplemental Fig. S4). To validate the binding specificity of apoE to the bacterial products, we treated apoE (5 μM) with LTA (200 μg/ml), which is the cell-wall product from Gram-positive *S. aureus*. We confirmed that apoE was not binding to LTA by not forming higher molecular complexes or not triggering changes in the secondary structure of the protein using electron microscopy, Blue Native gel, and CD (supplemental Fig. S5A–C).

**Fig. 7:**
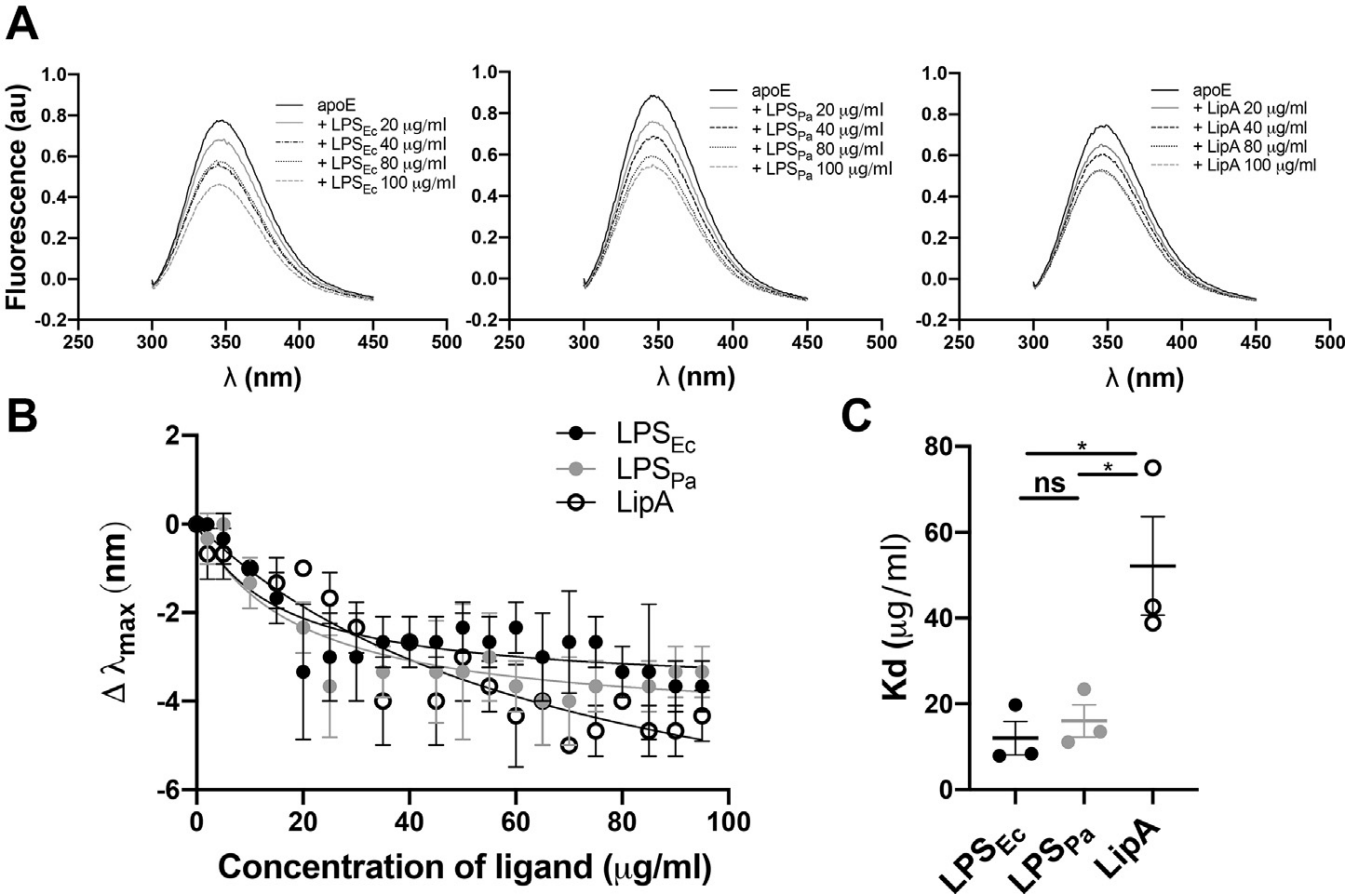
Structural property of apoE is affected by interaction with bacterial products. A: Increasing concentrations of LPS from *Escherichia coli* (LPS_Ec_), LPS from *Pseudomonas aeruginosa* (LPS_Pa_), or Lipid A from *E. coli* (LipA) were added to 5 μM apoE, and then, intrinsic fluorescence spectra were recorded. Figures show representative spectra from three independent experiments (n = 3). B: The binding curves were obtained reporting the shift in the λ_max_ of apoE alone compared with apoE in the presence of increasing concentrations of bacterial ligands (0–100 μg/ml). C: Dissociation constants (*K*_*d*_) of protein-ligand interaction were calculated from the binding curves. ∗*P* < 0.05. λ_max_, maximum absorbance; LPS, lipopolysaccharide; ns, not significant.

In addition, we used fluorescence spectroscopy to confirm the protein-ligand interaction. Tryptophan and tyrosine are relatively sensitive fluorophores, which are commonly used to detect structural properties of peptides and proteins (31). The microenvironment of the tryptophan amino acids in apoE was altered by the interaction with bacterial products. We observed fluorescence quenching and/or a spectral shift, which suggested tertiary structural changes in apoE after exposure to bacterial ligands (Fig. 7A). The shifts of wavelength of λ_max_ were analyzed to calculate *K*_*d*_ for LPS from *E. coli* (12.0 ± 3.9 μg/ml), LPS from *P. aeruginosa* (16.0 ± 3.7 μg/ml), and Lipid A from *E. coli* (52.3 ± 11.5 μg/ml) (Fig. 7B, C).

## Discussion

Research in the field of lipoproteins has the potential to discover new host defense molecules and the mechanisms by which they participate in the immune response to infection (12, 13, 23, 32). Several in vitro studies have shown that prototypic peptides with the sequence corresponding to the apoE receptor-binding region have strong antimicrobial activity (15, 16, 17, 19, 33). In addition, in vivo studies have revealed that apoE is a protein with anti-inflammatory (34, 35, 36, 37) and anti-infective properties, showing that apoE-deficient mice exhibited an increased susceptibility in response to Gram-negative or fungal infection (38, 39). Furthermore, it has been reported that apoE is an isoform-dependent antioxidant (40, 41, 42), and neuroprotective effects (20, 40) and epidemiological study on patient cohorts showed that the apoE level in the blood can be used as a biomarker to diagnose bacterial infection (35).

A major part of plasma apoE is associated with lipoproteins, but apoE also exists in a lipid-free form. The precursors for the cholesterol efflux pathway in vivo are likely to be lipid-free or lipid-poor apolipoproteins, which can be generated by dissociation from the surface of lipoproteins or by de novo synthesis (43). Moreover, apoE is expressed by skin cells in the epidermis, and apart from its role in blood, it can be regarded as a part of our innate defense system (44). Although the exact role of lipid-free apoE in the skin is not fully known, our findings suggest that apoE has a protective effect against Gram-negative bacteria.

To the best of our knowledge, the ability of the full-length apoE to directly affect bacteria or their endotoxins has not previously been reported. To address these points, we initially studied the antibacterial effect of intact apoE that was purified from human plasma. We demonstrated that the full-length protein affects Gram-negative bacterial survival in both in vitro and in vivo settings. This result could explain why apoE knockout mice were more predisposed to endotoxemia or *Klebsiella pneumoniae* or *Listeria monocytogenes* infection (45, 46).

Most of the previous studies have focused on the AMP derived from the receptor-binding region (amino acids 130–150 in ApoE). Thus, it has been reported that apoE-derived peptides (residues 133–149) show strong antimicrobial activity against Gram-positive and Gram-negative bacteria at the physiological concentration of apoE (1 μM). On the other hand, shorter apoE peptides (141–149) exhibited no antimicrobial activity at a similar concentration. It has been shown that amphipathic and cationic motifs are common structural features in AMPs and heparin-binding proteins and peptides (47). The observation that the antimicrobial activity of apoE was inhibited by heparin indicates that the heparin-binding region corresponding to residues 142–147 (48) mediates the effects of apoE on bacteria. The various antimicrobial and immunomodulatory activities of apoE-derived peptides are summarized in supplemental Table S1. Based on our in silico analyses, we have further substantiated and also extended previous reports on possible antimicrobial regions of apoE.

Moreover, we suggest that apoE induces bacterial killing by membrane disruption, which was the previously described mechanism of action for many classical AMPs (49, 50, 51). We also noticed that the bacteria treated with apoE aggregated, suggesting that apoE may be involved in the aggregation and clearance of toxic amyloid-β in the brain of patients with AD (52) and in the killing of microorganisms. The link between aggregation and antimicrobial activity is further supported by our previously published work on the host defense actions of proteolysed thrombin and its fragments, based on LPS-induced aggregation/scavenging and microbial killing (21, 25).

Furthermore, we investigated another aspect of apoE multifunctionality, such as the ability to interact with endotoxins, which could clarify the capability of apoE to neutralize the action of bacterial endotoxins. The structure of proteins is known to be affected by interaction with another ligands (53). For example, AMPs, which are commonly unstructured, tend to fold into a structured conformation upon interaction with endotoxins (54, 55). Therefore, we investigated if the structural properties of apoE were altered by binding to endotoxins. CD spectra showed a significant increase in the apoE α-helical content, confirming the changes in the secondary structure of the protein upon interaction with different bacterial products. Furthermore, by analyzing the intrinsic fluorescence of apoE, after incubation with endotoxins, we detected a decrease and a slight blue shift in the λ_max_ emission, suggesting that there was also a change in the protein’s tertiary structure. The presence of negative charges in LPS is essential for its biological activity (56). AMPs and LPS binding is characterized by hydrophobic and strong electrostatic interactions (57), which was also supported by our findings, analyzing binding constants (*K*_*d*_) using fluorescence spectroscopy to demonstrate that apoE binds to LPS from *E. coli* and *P. aeruginosa* with similar binding affinity. The interaction of apoE with Lipid A, which is the structural feature of LPS molecule responsible for the hydrophobic interaction (58), was approximately 4× lower. The difference in the binding affinity can be partially explained by the lack of the repetitive glycan polymer and the core oligosaccharide in the Lipid A molecules. Both structural features are present in LPS molecules, which provide a negative charge in addition to the hydrophobic core (59). Thus, negatively charged LPS is expected to have stronger binding affinity to positively charged apoE regions.

Finally, we found that in the presence of bacterial products from Gram-negative bacteria, apoE forms aggregates. The aggregate size was particularly smaller for apoE and Lipid A samples than LPS, which can be explained by the difference in the ligands’ molecular weight. This observation is also supported by our data from a previously published study on thrombin C-terminal-derived peptide (21), which similarly forms aggregates in the presence of bacteria and their endotoxins. Thus, the results suggest that aggregation-mediated clearance of bacterial infection is possibly a general mechanism for many amyloidogenic proteins with α-helical and β-sheet structural features (60), such as amyloid β peptides (61, 62), tau (63), and temporins (64).

Protein misfolding and aggregation are hallmarks in a broad range of amyloid-related diseases such as amyloidosis including Parkinson’s disease, Huntington’s disease, and AD. The exact mechanisms that cause protein aggregation remain unknown, but increasing evidence suggests that inflammation plays an important role in amyloid formation (65). Moreover, a previously published study showed that apoE4 isoform carriers may suffer a more excessive inflammatory response in sepsis, which links the apoE4 isoform to host defense and AD (66). Furthermore, amyloidogenic amyloid-β-peptides alone exhibit antimicrobial activity, which suggests that there is a connection between protein aggregation, host defense, and inflammation in patients with AD (67). Neutrophils have also been involved in the pathogenesis of amyloid deposits in the brain of patients with AD and AD mouse models (68). Hypothetically, apoE could lead to the formation of aggregates as a neutralizing action against bacterial infection in an environment with high inflammatory activity (69).

**Fig. 8:**
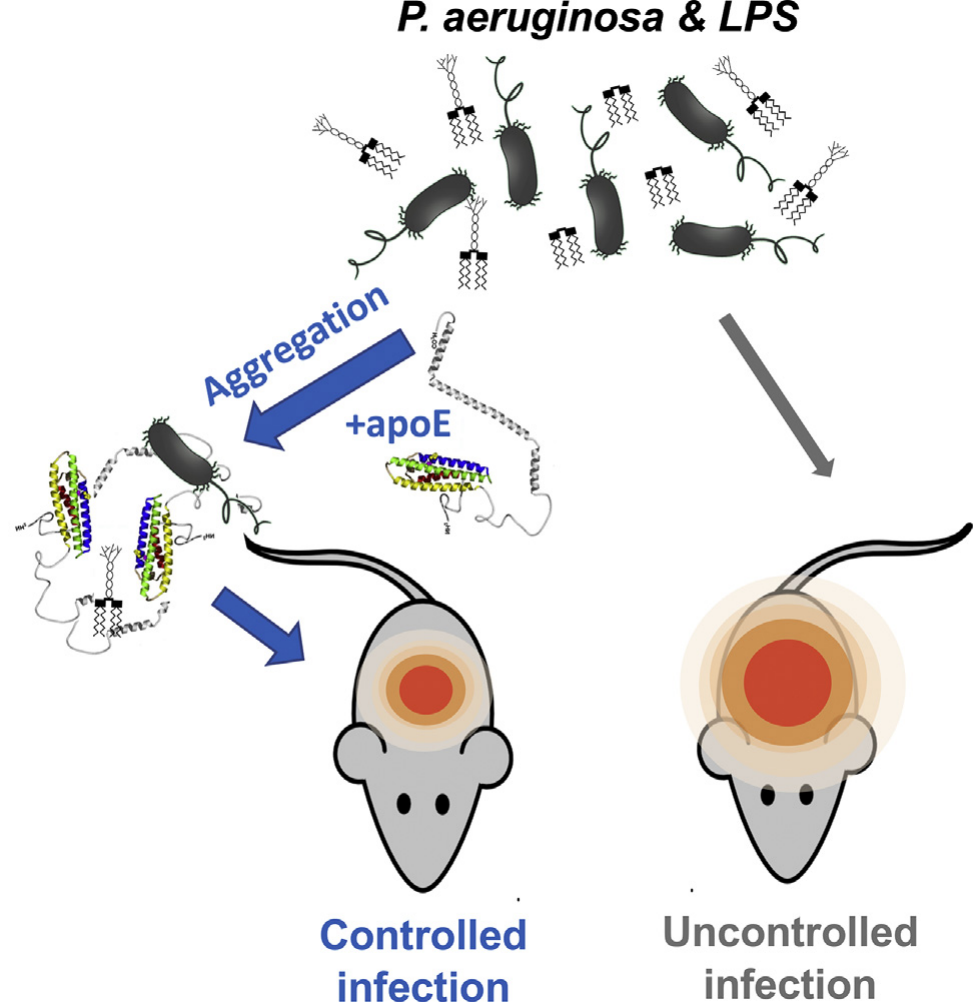
Summary of apoE function. Neutralizing effect of apoE on Gram-negative bacterial infection in vivo. The structure model of lipid-free apoE was published by Hatter *et al.* (70).

In conclusion, we have, for the first time, demonstrated that full-length apoE derived from human plasma has an antibacterial effect on *E. coli* and *P. aeruginosa*. In addition, we showed that plasma apoE has a different killing ability against *P. aeruginosa*, which was found to be more sensitive to the actions of apoE. The interaction of bacterial endotoxins with apoE was shown to affect the secondary and tertiary protein structures. Moreover, investigation of the apoE interaction with Gram-negative bacteria and their endotoxins revealed that apoE is likely to form aggregate-like complexes (Fig. 8). Taken together, these results offer a new perspective on the role of intact apoE proteins in the immunological response to bacterial infections and the development of new alternatives to antibiotics.

## Data availability

The authors declare that the data supporting the findings of this study are available within the article and its supplementary information files.

## Supplemental data

This article contains supplemental data.

## Conflict of interest

A. S. is a founder of in2cure AB, a company that is developing therapies based on thrombin-derived host defense peptides. The peptide GKY25 and variants are patent-protected. All other authors declare that they have no conflicts of interest with the contents of this article.
