# Supplementary material for: The role of full-length apoE in clearance of Gram-negative bacteria and their endotoxins
Source: J Lipid Res. 2021 May 18;62:100086. doi: 10.1016/j.jlr.2021.100086 (PMC8225977; doi:10.1016/j.jlr.2021.100086)
Supplement: Supplemental Figures S1–S5 and Table S1 [file mmc3.docx]

**Supplemental Information:**

#

# The role of full-length apolipoprotein E in clearance of Gram-negative bacteria and their endotoxins

Ganna Petruk^1#^, Malin Elvén^1#^, Erik Hartman^1^, Mina Davoudi^1,2^, Artur Schmidtchen^1,3,4^, Manoj Puthia^1^ and Jitka Petrlova^1*^

^1^Division of Dermatology and Venereology, Institution of Clinical Sciences, Lund University, SE-22184 Lund, Sweden

^2^Division of Cancer and Infection Medicine, Institution of Clinical Sciences, Lund University, SE-22184 Lund, Sweden

^3^Copenhagen Wound Healing Center, Bispebjerg Hospital, Department of Biomedical Sciences, University of Copenhagen, DK-2400 Copenhagen, Denmark

^4^Dermatology, Skane University Hospital, SE-22185 Lund, Sweden

**Supplementary Figure S1.** Effects of heparin on apoE **A)** *P. aeruginosa* was incubated for 2 h with 5 µM apoE and heparin (pre-mixed for 30 min) followed by analysis by viable count assay. Data are presented as the mean ± SEM of three independent experiments (n=3). Statistical analysis was performed using a one-way ANOVA with Dunnett’s multiple comparison tests, * = p ≤ 0.05, *** = p ≤ 0.001. **B)** Far-UV circular dichroism spectra of 5 µM apoE with 200 and 500 µg/ml heparin. Figures show representative spectra from at least three independent experiments (n ≥3).

**Supplementary Figure S2 A)** Helical wheel of the common peptide sequence (LRVRLASHLRKLRKRLL). The amphipathic helical wheel shows two distinct regions of hydrophobic and hydrophilic residues. **B)** 3D model of apoE and the position of the peptides, which are indicated in the NMR structure of human apoE (PDB code 2L7B), with the highest antimicrobial score.

**

**Supplementary Figure S3. A)** ApoE (5 μM) was incubated with 200 μg/ml LPS from *E. coli* or *P. aeruginosa* (denoted as LPS_Ec_ and LPS_Pa,_ respectively) or with Lipid A from *E. coli* (LipA) for 60 min at 37°C. At the end of incubation, the macromolecular complexes were visualized by TEM. Figures show one representative image for each experiment (n=3). Scale bar represents 1 µm. **B)** ApoE (5 µM) or apoA1 were mixed with either Tris buffer or 200 μg/ml LPS from *E. coli* (LPS_Ec_) and incubated for 30 min at 37°C and then analyzed by Blue Native PAGE. One representative image from three independent experiments is shown (n=3). **C)** Far-UV circular dichroism spectra of 5 µM apoA1 and apoE with 100 µg/ml LPS from *E. coli*. Figures show representative spectra from at least three independent experiments (n ≥3).

**Supplementary Figure S4.** Far-UV circular dichroism spectra were recorded of 5 µM plasma apoE alone or after adding 200 µg/ml LPS from *E. coli* (LPS_Ec_), LPS from *P. aeruginosa* (LPS_Pa_), or Lipid A from *E. coli* (LipA) at 25ºC. α-Helical content calculated from molar ellipticity at 222 nm (**[θ]**222). Statistical analysis with Brown–Forsythe ANOVA analysis was performed using GraphPad Prism. Bars represent the mean ± SD from at least three independent experiments (n ≥3). **** = p<0.0001.

**Supplementary Figure S5. A)** ApoE (5 μM) was incubated with 200 μg/ml LTA from *S. aureus* (LTA_Sa_) or Tris buffer for 60 min at 37°C. At the end of incubation, the macromolecular complexes were visualized by TEM. One representative image for each condition from three independent experiments is shown (n =3). Scale bar represents 1 µm. **B)** ApoE (5 µM) was mixed with either Tris buffer or 200 μg/ml LPS from *E. coli* (LPS_Ec_) or 200 μg/ml LTA from *S. aureus* (LTA_Sa_) and incubated for 30 min at 37°C and then analyzed by western blot following Blue Native gel. One representative image from three independent experiments is shown (n=3). **C)** One representative Far-UV circular dichroism spectra of 5 µM apoE with 200 µg/ml LTA from *S. aureus* (LTA_Sa_) (n =3).

| **Region** | **Peptide** | **Activity** | **Ref.** |
| --- | --- | --- | --- |
| 130–149 | TEELRVRLASHLRKLRKRLL | Decreased airway hyperresponsiveness, airway inflammation, and mucous cell metaplasia in Apoe-deficient mice.  Bind to LRP1 with high affinity. | **(1)**  **(2)** |
| 133-149 | LRVRLASHLRKLRKRLL | Reduce infectivity of HSV-1, HSV-2 and HIV (antiviral). Antibacterial against *P. aeruginosa and S. aureus.*  Reduce CNS and systemic inflammation.  Suppress microglia activation. | **(3)**  **(4)**  **(5)** |
| 133–150 | LRVRLASHLRKLRKRLLR | Broad spectrum antibacterial. Anti-inflammatory effect on human keratinocytes. | **(6)** |
| 133-162 | LRVRLASHLRKLRKRLLRDADDLQKRLAVY | Antibiotic activity towards G- and G+ bacteria. | **(7)** |
| 133-167 | LRVRLASHLRKLRKRLLRDADDLQKRLAVYQAGA | Broad spectrum antibacterial. Downregulate LPS-induced cytokine release. Antibiofilm properties. | **(8)** |
| 141-149 | LRKLRKRLL | Mildly antibacterial. | **(3)** |
| 141–155 | LRKLRKRLLRDADDL | Inhibit LPS and poly(I-C)-induced macrophage inflammatory responses. | **(9)** |
| 263-286 | SWFEPLVEDMQRQWAGLVEKVQAA | Mildly antibacterial. | **(3)** |

**Supplementary Table S1. Published apoE derived peptides and their activities.**

**References**

1. Yao, X., Fredriksson, K., Yu, Z. X., Xu, X., Raghavachari, N., Keeran, K. J., Zywicke, G. J., Kwak, M., Amar, M. J., Remaley, A. T., and Levine, S. J. (2010) Apolipoprotein E negatively regulates house dust mite-induced asthma via a low-density lipoprotein receptor-mediated pathway. *Am J Respir Crit Care Med* **182**, 1228-1238

2. Croy, J. E., Brandon, T., and Komives, E. A. (2004) Two apolipoprotein E mimetic peptides, ApoE(130-149) and ApoE(141-155)2, bind to LRP1. *Biochemistry* **43**, 7328-7335

3. Dobson, C. B., Sales, S. D., Hoggard, P., Wozniak, M. A., and Crutcher, K. A. (2006) The receptor-binding region of human apolipoprotein E has direct anti-infective activity. *J Infect Dis* **193**, 442-450

4. Laskowitz, D. T., Fillit, H., Yeung, N., Toku, K., and Vitek, M. P. (2006) Apolipoprotein E-derived peptides reduce CNS inflammation: implications for therapy of neurological disease. *Acta Neurol Scand Suppl* **185**, 15-20

5. Laskowitz, D. T., Thekdi, A. D., Thekdi, S. D., Han, S. K., Myers, J. K., Pizzo, S. V., and Bennett, E. R. (2001) Downregulation of microglial activation by apolipoprotein E and apoE-mimetic peptides. *Exp Neurol* **167**, 74-85

6. Pane, K., Sgambati, V., Zanfardino, A., Smaldone, G., Cafaro, V., Angrisano, T., Pedone, E., Di Gaetano, S., Capasso, D., Haney, E. F., Izzo, V., Varcamonti, M., Notomista, E., Hancock, R. E., Di Donato, A., and Pizzo, E. (2016) A new cryptic cationic antimicrobial peptide from human apolipoprotein E with antibacterial activity and immunomodulatory effects on human cells. *FEBS J* **283**, 2115-2131

7. Azuma, M., Kojimab, T., Yokoyama, I., Tajiri, H., Yoshikawa, K., Saga, S., and Del Carpio, C. A. (2000) A synthetic peptide of human apoprotein E with antibacterial activity. *Peptides* **21**, 327-330

8. Zanfardino, A., Bosso, A., Gallo, G., Pistorio, V., Di Napoli, M., Gaglione, R., Dell'Olmo, E., Varcamonti, M., Notomista, E., Arciello, A., and Pizzo, E. (2018) Human apolipoprotein E as a reservoir of cryptic bioactive peptides: The case of ApoE 133-167. *J Pept Sci* **24**, e3095

9. Zhu, Y., Kodvawala, A., and Hui, D. Y. (2010) Apolipoprotein E inhibits toll-like receptor (TLR)-3- and TLR-4-mediated macrophage activation through distinct mechanisms. *Biochem J* **428**, 47-54
